# Personal Variables in Attitude toward Green Purchase Intention of Organic Products

**DOI:** 10.3390/foods13020213

**Published:** 2024-01-10

**Authors:** Hector Juan Palomino Rivera, Luciano Barcellos-Paula

**Affiliations:** CENTRUM Catolica Graduate Business School, Lima, Perú—Pontificia Universidad Católica del Perú, Lima 64032, Peru; lbarcellosdepaula@pucp.edu.pe

**Keywords:** green purchasing intention, organic products, environmental awareness, green self-identity, subjective norms, environmental attitude

## Abstract

The present research aims to determine whether environmental awareness, green self-identity, and subjective norms influence the attitudes of consumers who identify with environmental issues and have green purchasing intentions for organic products. The research was quantitative, correlational in scope, and cross-sectional in design. It was applied to 710 Peruvian millennials. A questionnaire consisting of 20 questions was applied, which was quantified through a five-point Likert scale. The results were processed through an Exploratory Factor Analysis (EFA), a Confirmatory Factor Analysis (CFA), and Structural Equation Modeling (SEM). Statistical analyses were developed using SPSS 24 and AMOS 24. The study identified that the personal variables influencing the environmental attitudes of millennials who intend to buy green organic products are green self-identity and subjective norms. While environmental awareness does not influence environmental attitudes, it does influence the green self-identity of Peruvian millennials. This study is one of the first to identify the personal variables influencing the environmental attitudes of Peruvian millennials who intend to buy green organic products.

## 1. Introduction

Over time, climate variation, air and water pollution, and waste generation have become topics of social discussion regarding environmental care [1]. As a result, the protests of environmental collectives have motivated global organizations to adopt measures to safeguard the planet [2,3,4]. In this regard, the United Nations (UN) has been proposing measures to solve this problem [5], one of the most important of which is the incorporation of the Sustainable Development Goals (SDGs), point 12 of which refers to the need to reduce our ecological impact through the implementation of environmentally friendly production and responsible consumption, thus ensuring compliance with a target of the SDGs which determines that by 2030, it is necessary to ensure that people worldwide are aware of sustainable development and lifestyles that are in harmony with nature.

Due to the fact that the excessive consumption of products and the lack of awareness of environmental issues have had an impact on the degradation of the environment in recent years [6,7], the consumption of organic products has become an option that allows consumers to satisfy their needs with the least possible impact on the ecosystem [1,8,9,10,11,12,13]. According to Ostapenko et al. [14], organic products are developed without chemical elements that alter their production and generate environmental deterioration. Within the field of consumer behavior, several research studies have been developed over time to identify the drivers that encourage consumers to purchase environmentally identified products [4,13,15], determining that purchase intention for organic products is derived from consumers’ attitudes toward environmental issues [3,16,17,18]. Therefore, attitude has been defined as a consumer’s positive or negative valuation of a behavior [19].

This study examines behaviors that should be adopted to not negatively impact the environment [20,21]. In view of this, several studies have determined that consumers with positive attitudes toward organic food believe that buying this type of product is important and a good option for them [4,13]. The study by Kumar et al. [22] found that a favorable attitude towards environmental issues influences the intention to purchase organic products. At the same time, Jaiswala and Kant [5], through their study, determined that consumers are stimulated by cognitive factors that directly and indirectly influence green purchase intention through the mediating role of attitude. On the other hand, Taufique and Vaithianathan [3] determined that attitudes towards the environment significantly directly and positively influence green purchase intention and environmentally conscious consumer behavior. Moreover, recent studies, through their hypothetical models, have further corroborated this relationship, determining that attitude is the factor that has the highest correlation with purchase.

Public concerns about environmental issues and the need to understand the factors influencing purchasing behavior that is aligned with sustainability have become topics of high interest for the academic community and the food production sector [17,18,23]. Although several research studies have identified that attitude is a major element influencing purchase intentions for organic products [1,9,13,21,24,25], some researchers have identified the existence of knowledge gaps regarding the lack of knowledge of the factors that influence attitude as a determinant of purchase intentions for organic products [20,26,27]. Other studies have stated that the need to understand the factors that lead to purchase intentions for environmentally identified products within emerging countries is still pending [25,28,29,30], while Hoyos et al. [21] determined that there is a need to develop research models that analyze the influence of environmental awareness, green self-identity, and subjective norms within attitudes towards green purchase intentions.

In turn, within the academic field, there has been a need to determine the factors that influence the green purchasing behavior of millennials. This assertion is supported by Carrión et al. [13], who determined that it is worth studying in depth the consumption preferences of university students. Millennials are considered an appropriate study segment, since their actions are geared towards environmental protection, they are able to purchase organic products, and they influence their environment to adopt similar purchasing decisions [4].

Against the above theoretical background and to expand theoretical knowledge about the factors that influence attitude to be a determining factor in the purchase intentions of organic products, this study was developed under a quantitative research approach, is correlational in scope, and uses a cross-sectional design, and through the application of a questionnaire, it allowed us to answer the research question: What are the personal variables that influence millennials’ attitude towards the intention to buy green organic products?

In order to answer the central research question, What are the personal variables that influence millennials’ attitude towards the intention to purchase green organic products, the present study aimed to determine whether factors such as (a) environmental awareness, (b) green self-identity, and (c) subjective norms influence the attitudes of consumers who identify with environmental issues and have green purchasing intentions for organic products. The study consists of the following sections: (1) introduction, (2) literature review, (3) methodology, (4) results, (5) discussion, (6) conclusion, (7) implications, and (8) limitations and recommendations for future research.

## 2. Literature Review

### 2.1. Green Purchasing Intention

Green purchase intention (GPI) refers to a consumer’s future predisposition to purchase products that are aligned with environmental protection [13]. Likewise, for Sheng [31], GPI is the prelude to behavior and refers to a conscious action plan that allows an individual to reach a specific goal. Considering that intention is the preliminary step to purchase, many researchers have identified that attitude is one of the main factors influencing the intention to purchase an organic product [4,21,32].

The academic literature has used words such as “green consumption”, “adoption of ecological or organic products”, or “green purchasing” to describe the different purchasing behaviors that are aligned with the protection of the environment [6,13]. Green consumption refers to the pro-environmental attitude and awareness of environmental problems [33]. For Liobikiene and Bernatoniene [2], this type of consumption does not focus on decreasing the acquisition of products by consumers; its main objective is to reduce the environmental impact.

Over time, several studies have agreed that environmental protection has led consumers to support green consumption actively [13,27,34]. However, there is a large disconnect between green purchase intention and actual purchase behavior [4]. The discrepancy or gap between consumers’ favorable attitude towards the environment and actual purchase behavior is referred to as the “green purchase inconsistency” or “green behavioral attitude gap” [13]. For this reason, today’s companies must adapt to the competitive demands of the contemporary market and think in a “greener” way [21].

On the other hand, millennials population is considered the largest generation of consumers worldwide, and their habits are characterized by their preference for products that are aligned with the environment [4]. Millennials are considered an appropriate study segment, since their actions are geared towards environmental protection, they have the possibility to buy organic products, and they generate advocacy in their environment to encourage others to adopt similar purchasing decisions, which can be corroborated through the interest placed in this area [13].

### 2.2. Environmental Awareness

EA is one of the vital cognitive constructs in predicting green behaviors [20]. For Jaiswal and Kant, environmental concerns are positively connected to purchase intentions for organic products. According to Bülbül et al. [35], EA consists of two dimensions: (a) the sensitivity dimension and (b) the willingness dimension. In the sensitivity dimension, consumers are quite sensitive to environmental issues; that is, they not only intend to purchase organic products but also recycle and reduce energy consumption, among other activities aligned with environmental protection [36]. The willingness dimension refers to the predisposition to acquire consumption behaviors that are aligned with environmental protection despite the high prices of products and their availability [35].

Several studies have tested the relationship of EA with purchase intentions for organic products, such as the study by Suárez et al. [37], who analyzed the role of EA in pro-environmental behaviors and concluded that EA does not need to be translated into personal actions to preserve the environment. On the other hand, Shelest et al. [38] indicated that EA is an important predictor of pro-environmental behaviors. At the same time, Aliman and Astina [39] indicated that EA motivates people to behave eco-protectively. Regarding EA among university students, Hansman et al. [40] examined the determinants of environmental protective behavior and determined a positive connection between EA and environmental protective behavior. The study concluded that the higher the level of EA, the more the individual will be concerned about environmental disputes and, therefore, engage in green behaviors. On the other hand, Carducci et al. [41] found that a higher level of EA causes a person to engage in different environmentally friendly and climate-friendly behaviors. The study by Bülbül et al. [35] argued that environmental pollution can be controlled by spreading awareness of different environmental and climate change issues among people. This awareness, in turn, causes individuals to behave environmentally friendly, consequently improving environmental quality [36]. The academic literature has shown that some studies have included EA within extended frameworks that have sought to identify its influence on green consumption [20]. However, the literature review provided evidence that the level of influence that this variable has on the EA and GSI of millennials who intend to purchase organic products have not been tested. In consideration of the above, the following hypotheses are put forward:

 **Hypothesis** **1** **(H1).**  *Environmental awareness positively influences the environmental attitude of millennials.*

 **Hypothesis** **1a** **(H1a).**  *Environmental awareness positively influences the green self-identity of millennials.*

### 2.3. Green Self-Identity

Self-identity is the collection of roles enacted by a person, which results in consistent action within their behavior [42]. Self-identity is the group of principles that each person has, which can induce the performance of some actions [43]. Following that logic, it is considered that an individual can have a GSI when he/she identifies with the environment, and this encourages him/her to have an environmental awareness and thinking attitudes that align with buying organic products [44]. According to Kumar [22], from an epistemological point of view, GSI is made up of two dimensions: (a) emotion and (b) cognition. The emotion dimension refers to the level of empathy shown by consumers to prevent the deterioration of the planet.

Meanwhile, the cognitive dimension is about the level of knowledge that a person has regarding the environmental degradation that occurs with each of the daily activities of human beings [42]. Several studies related to green consumption have incorporated GSI within their research models [29,43], which have determined that a common factor within this research is that an individual’s self-perception can be an important determinant of green purchasing behaviors [45]. For example, Whitmarsh and O’Neill [46] identified that GSI is related to the intention to buy organic products.

Moreover, Khare [47] stated that the positive impact of GSI influences the intention to purchase environmentally friendly products, while Barbarossa and De Pelsmacker [44] found that GSI is an antecedent of the intention to buy green products. Research such as that by Confente et al. [48] found that GSI leads consumers to generate perceptions of the value of environmentally identified products, directly and significantly influencing the intention to purchase bioplastic products. Sharma et al. [29] explored the impact of GSI on GPI and concluded that a consumer’s self-identity significantly impacts the intention to purchase organic products. Despite the existence of a wide variety of research on the influence of GSI on purchase intentions, a review of the literature revealed that there are no studies that determine whether this variable influences the environmental attitudes of millennials to promote the intention to buy organic products, which leads to the following hypothesis:

 **Hypothesis** **2** **(H2).**  *Green self-identity positively influences the environmental attitude of millennials.*

### 2.4. Subjective Norms

SNs means social norms that are conceived as society’s influence on the development of a certain behavior [4,49]. This construct results from two dimensions: (a) normative belief and (b) motivation to comply. Normative belief refers to the individual’s perception of how other people want an individual to behave in a given situation, while compliance motivation refers to the individual’s desire to comply with the opinion of others [19]. Empirical evidence suggests a strong association between SNs and many pro-environmental behaviors, including the intention to purchase organic products [4,5].

According to Ricci et al. [50], the academic literature on green behavior supports the determination that these norms can play an extremely important role due to the degrees of social influence that they exert on behavior. Furthermore, several studies reveal that young consumers tend to consider the opinions and expectations of those who are considered important to them, such as friends, family, and colleagues, when engaging in pro- environmental behavior [2,4,51,52,53]. Research by Wang et al. [34] indicated that SNs exert a significantly positive influence on green purchases. Moreover, individuals who are motivated to purchase environmentally identified products do so because they have received positive references about green products [2], thus proving that consumers’ close environments influence purchase intentions [5,21,53].

Although the literature in favor of SNs is clear [2,34,51,52,53], and recent research has proven that these norms influence millennials’ green purchase intentions [4,13], other research has questioned the role of these norms within green purchase intentions [54]. According to Thogersen and Zhou [55], SNs play no role in predicting purchase intention for organic products such as organic food in China, while Paul et al. [56] and Kumar et al. [22] established that these standards do not significantly predict purchase intention. On the other hand, Taufique and Vaithianathan [3] found that the incidence of SNs is insignificant in purchase intention.

Although these norms imply a proper feeling of social pressure towards certain behaviors, their influence on green consumption is not yet confirmed [33], in the face of which some research has questioned their role in shaping the attitudes of environmentally identified individuals [54], as is the case of Kumar et al. [22], who suggested that SNs do not play any role in predicting purchase intention for green products such as organic food. On the other hand, Taufique and Vaithianathan [3] found that social norms (SNs) are insignificant concerning the direct effect on behavioral intention. Although these norms imply an inherent social influence on certain behaviors, their influence on green consumption is not yet confirmed [33]. Based on the above, and considering the scarce literature that determines whether social norms influence the EAT and GSI of millennials who have green purchase intentions, the following hypotheses are proposed:

 **Hypothesis** **3** **(H3).**  *Subjective norms positively influence the environmental attitude of millennials.*

 **Hypothesis** **3a** **(H3a).**  *Subjective norms positively influence the green self-identity positively of millennials.*

### 2.5. Environmental Attitude

Attitude is a consumer’s positive or negative appraisal of a behavior [5]. According to Ajzen and Fishbein [49], attitude consists of two dimensions: (a) behavioral belief, which refers to an individual’s recognition of the consequences of engaging in a particular behavior, and (b) outcome evaluation, which refers to an individual’s favorable or unfavorable judgment of the possible consequences of a behavior. Several authors have stated that EAT is one of the strongest predictors of green consumption. According to Jaiswala and Kant [5], their study determined that consumers are stimulated by cognitive factors that indirectly influence GPI through the mediating role of attitude. At the same time, Taufique and Vaithianathan [3] determined that EAT has a significantly direct and positive influence on GPI and ecologically conscious consumer behavior.

Moreover, recent studies have proven that EAT significantly influences purchase intentions for organic products [4,13,53]. Although the academic literature supports the argument that environmentally friendly attitudes have been central to the understanding of environmentally friendly behavior and their influence on purchase intention is evident [17,57], some researchers still question the role of attitude in consumer studies, determining that it has not been fully addressed within GPI [54]. In consideration of the above, the following hypothesis is put forward:

 **Hypothesis** **4** **(H4).**  *Environmental attitude positively influences the green purchase intention of millennials.*

**Figure 1:**
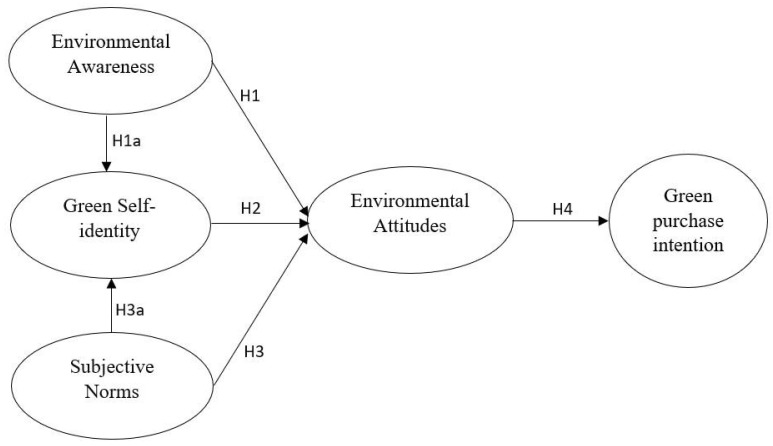
Research hypothesis model.

The hypothesized research model is presented below (See Figure 1).

## 3. Methodology

The study was quantitative, correlational in scope, and cross-sectional in design. Data for the study were collected through a survey of 25 questions (5 demographic questions and 20 for the hypothesized model variables), which were measured using a five-point Likert scale. The questions to measure the study variables were taken from previous research on green consumption (See Appendix A). The questionnaire was validated through a panel of experts made up of research and marketing specialists, and subsequently, a pilot test was developed with 30 millennials.

The study population was Peruvian millennials living in the city of Lima. This population cohort was considered because the literature on green consumption has determined that millennials are the population that most identify with environmental issues, and their purchasing behavior is aligned with the consumption of organic products [13]. The survey was applied outside shopping centers, and the sampling was probabilistic, through which 731 participants who freely and voluntarily decided to participate in the research were selected. A total of 20 surveys were discarded due to inconsistencies in their completion. Therefore, statistical analyses were processed with 710 participants.

Demographic characteristics of respondents:

The study was carried out in the city of Lima, Peru. A total of 710 surveys were used for statistical analysis. To balance the study sample, the three population sub-cohorts of millennials [13] and millennials of the male and female gender who are studying or have completed undergraduate and postgraduate studies were considered (see Table 1).

### 3.1. Internal Consistency of the Instrument

Statistical analyses were developed using recently published articles on green consumption as a guide [13,21]. A Cronbach’s Alpha test was developed to analyze the consistency of the applied questionnaire. The internal consistency analysis determined that it was necessary to eliminate the relative value of the *X*^2^ divided by the degrees of freedom (*X*^2^/df), which was initially determined to check the goodness-of-fit indices of 0.7, as established in the literature [13,58,59].

### 3.2. Exploratory Factor Analysis (EFA)

The Kaiser–Meyer–Olkin (KMO) test yielded a value of 0.900 and a significance level of 0.001 (*p* < 0.05), values that are accepted by the academic community [13,60]. Likewise, the variance explained showed that the study items were grouped into five dimensions with a percentage of 71.83%, a value that exceeds the 60% recommended by Streiner [61]. On the other hand, the rotated components matrix was used to check that the questions were grouped into their corresponding dimensions, which showed that the items were correctly grouped within their constructs.

### 3.3. Data Analysis

A Confirmatory Factor Analysis (CFA) was performed to measure the convergent and discriminant validity of the hypothesized model, while the hypotheses were tested through Structural Equation Modeling (SEM) developed in AMOS 24. To check the goodness-of-fit indices, the relative value of the *X*^2^ divided by the degrees of freedom (*X*^2^/df) was initially determined. When the fit is less than 3.0, the model is acceptable [13,21,58,59,62]. Next, the goodness-of-fit index (GFI), the comparative fit index (CFI), the Tucker–Lewis index (TLI), and the incremental fit index (IFI) were calculated, where values above 0.95 indicate an excellent fit [58]. Finally, the following values were determined: the residual root mean square (RMR) and the root mean square error of approximation (RMSEA), where values below 0.80 indicate that the data have a good fit [4,58].

## 4. Results

### 4.1. Estimation of the Research Model

The hypothesized model, which was composed of five variables (environmental awareness, green self-identity, subjective norms, environmental attitude, and green purchase intention), was tested through a CFA. To determine the existence of convergent validity, it is necessary to meet the following values: Cronbach’s Alpha ≥ 0.70; Composite Reliability (CR) ≥ 0.70; and Average Variance Extracted (AVE) ≥ 0.50 [13,21,63,64,65]. Hair et al. [59] also indicated that when the AVE values are lower than the CR values, the existence of convergent validity is further proven (see Table 2).

Table 3 shows the test for discriminant validity. We first calculated the bivariate correlational values of the constructs of the hypothesized model and then calculated the Square Root of each AVE value (SR AVE). When the SR AVE values are greater than the values of the correlations of each of the constructs, the discriminant validity of the model is corroborated [13,21,64,66].

### 4.2. Structural Model: Model Fit and Hypothesis Testing

Once the hypothesized model’s convergent and discriminant validity criteria had been tested, an SEM was developed to approve or reject the study’s hypotheses. Statistical analyses tested the relationships between the five variables of the model, and the results derived from the maximum likelihood estimation developed in AMOS 24 showed that the data met the following goodness-of-fit indices: *X*^2^ (gl) = 344. 243 (129); *X*^2^/g = 2.669; NFI = 0.960; TLI = 0.969; CFI = 0.974; root mean squared error of approximation (RMSEA) = 0.048 [13,58,59].

**Figure 2:**
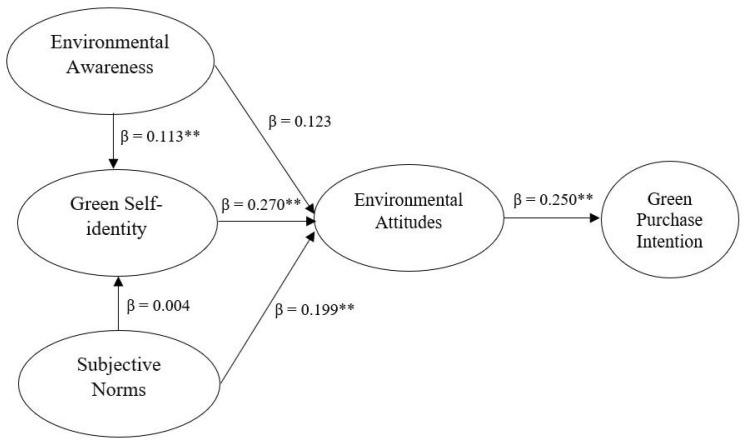
Values in the hypothesized model. ** The correlation is significant at the 0.01.

Once the relationships between the five variables of the hypothesized model were analyzed and considering the significance values (*p* < 0.05), four hypotheses were accepted, and two were rejected. That is, the estimated values determined that EA does not influence EAT (*β* = 0.123; *p* > 0.05) but does influence GSI (*β* = 0.113; *p* < 0.05). Likewise, GSI was proven to influence EAT (*β* = 0.270; *p* < 0.05). Regarding SNs, analyses determined that SNs influence EAT (*β* = 0.199; *p* < 0.05) but do not influence GSI (*β* = 0.004; *p* > 0.05). Finally, EAT influenced the GPI of Peruvian millennials (*β* = 0.250; *p* > 0.05), see Table 4 and Figure 2.

## 5. Discussion

Identifying factors influencing organic product consumption is a topic that has become relevant in today’s academic and business contexts. Although recent studies within the South American context have found inconsistency gaps between millennials’ attitudes and green purchasing intentions [4,13], the present study provides valuable information indicating the motivating factors that influence the attitudes of millennials who intend to purchase organic products.

Considering the above, the research model analyzed in this study allowed us to reaffirm the influence of attitude on intentions to purchase organic products and, in turn, allowed us to identify the personal factors that stimulate the environmental attitudes of millennials who intend to purchase products that are aligned with the environment. Statistical analyses allowed us to determine that H1 is rejected. This means that EA does not influence the EAT of millennials. This means that Peruvian millennials believe that it is not necessary to achieve social or political changes to improve consumer awareness and that it is not necessary to apply severe laws against pollution, since consumers should assume their own responsibility to protect the environment. Despite the lack of studies that have tested the relationship between EA and EAT, the findings evidenced through this hypothesis support Suárez et al. [37], who determined that EA need not manifest itself through personal actions in favor of the consumption of products that are aligned with environmental protection and contradict several investigations that have determined that EA is an important predictor that stimulates GPI [36,38,41].

On the other hand, the study identified an important finding that explains the lack of a relationship between EA and EAT, showing that although EA does not influence EAT, it does impact GSI. This means that EA positively influences the GSI of Peruvian millennials, which is why H1a is accepted. This means that Peruvian millennials do not need EA to have pro-environmental attitudes, but they do need it to consider themselves green consumers, and that the satisfaction of consuming organic products influences their GSI. This finding supports research findings that consumers’ awareness of environmental pollution increases their green identity and makes them more sensitive to purchasing organic products [36], and that the greater the environmental concern is, the greater the self-identity for environmental protection is [48,67].

H2 is accepted. This means that GSI positively influences the EAT of millennials. This means that Peruvian millennials consider themselves to be green consumers, and that their inclination towards organic products influences their identity and, therefore, the increase in their EAT and the belief that environmental protection is important when consuming a product. Although the literature on green consumption does not present studies that relate GSI to EAT, the finding generated through this hypothesis supports the position of research that shows that GSI is a factor that promotes a consumer’s positive actions, stimulating their attitude in favor of environmental protection [29,44,46,48], and that GSI stimulates a consumer’s reason [42] and encourages purchasing behaviors that are aligned with environmental conservation [44,68].

Concerning the SNs, the statistical analyses of the study allowed us to accept H3, which posits that SNs positively influence millennials’ EAT. This finding shows that the influence of Peruvian millennials’ friends, family, and colleagues affects their EAT, confirming that the approval of their social circles conditions their attitudes in favor of environmental protection. This finding corroborates the findings of several investigations that have identified the influence of SNs on millennials’ attitudes [2,13,34,50,53], as well as studies that determined that the influence of family and friends impact attitudes [2,4,51,53] and contrasts with positions of researchers who still question the influence of SNs on consumer attitudes [3,22,33,54].

On the other hand, the study did not prove the positive influence of SNs on the GSI of millennials. Therefore, H3a is rejected, which shows that the opinions of friends and family on the purchase of organic products does not influence the generation of GSI of Peruvian millennials. This contradicts Hui and Khan [69], who found that SNs mediate the relationship between GSI and consumer attitudes. Finally, the study tested the relationship between EAT and GPI, whereby H4 is accepted. This means that EAT positively influences GPI, showing that Peruvian millennials consider organic products to help save nature, and their consumption preferences are aligned with purchasing organic products instead of conventional ones. This finding supports the determination that millennials’ attitudes significantly influence LCIs [4,13,17,57] and contradicts research that has questioned the role of EAT in GPI, such as that by Testa et al. [54], who indicated that the role of attitude in GPI has not been fully tested, as well as Tang et al. [7], who found that consumer attitudes do not have a direct impact on GPI.

## 6. Conclusions

The present study took into consideration the recommendations of studies that stated that the factors that influence attitude as a determining factor in green purchase intentions are not fully understood, and to answer the research question “What are the personal variables that influence the attitude towards the intention to purchase organic products of millennials?”, it proposed a research model that allowed the following findings to be obtained: (a) EA does not influence the EAT, however, it does influence the GSI, (b) the GSI influences the EAT, and (c) SNs influence the EAT but do not influence the GSI of Peruvian millennials. Considering the above, it is concluded that the personal variables influencing the EAT of millennials with GPI are the GSI and SNs.

The study has theoretical, practical, and social implications. From a theoretical point of view, the study’s findings reinforce the academic literature that states that attitude is one of the factors that considerably influences GPI and provides new academic support regarding the personal variables that influence EAT. Likewise, having determined that SNs influence Peruvian millennials’ attitudes allows us to reduce the gap concerning the differences in criteria that some researchers have regarding the influence of SNs on GPI. From a practical point of view, the study provides valuable contributions to the business field, since it provides information that allows companies to understand in greater depth the drivers that stimulate GPI and thus propose marketing plans to strengthen the GSI and EAT of consumers and in other cases to raise awareness so that millennials who do not identify with the consumption of organic products see in this type of product an opportunity to contribute to environmental protection. Finally, from a social point of view, the study provides information that shows that EA generates GSI, which increases EAT and GPI. Because of this, a call is made to governmental organizations and educational institutions in Peru to develop actions to increase the EA of Peruvian consumers and the consumption of organic products and thus contribute to the fulfillment of the Sustainable Development Goals proposed by the United Nations and the goal that determines that by 2030, the world population should know about sustainable development and lifestyles that are aligned with nature.

As part of the limitations of the study, it was determined that having included millennials in the city of Lima, Peru, as the study population limits the generalizability of the study’s results to all millennials in the country. On the other hand, the fact that the survey was conducted outside shopping centers may lead to a purchasing behavior bias, since it did not consider millennials who also intend to buy organic products but do not necessarily go to shopping centers to buy this type of product. Several studies have shown that self-reported questionnaires can lead to the presence of untruthful statements by respondents [4,70,71], so some of the millennials may not have answered the questions in the questionnaire truthfully. In consideration of the above limitations, it is recommended that future research should develop studies on GPI by equating the study sample with participants from all provinces of Peru. It is also necessary that future studies not only consider millennials within their study samples but also consider other population cohorts such as baby boomers, generation X, and centennials. To eliminate information biases produced through self-reported questionnaires, it is recommended that future research should develop qualitative studies through in-depth interviews that identify the determinants that influence consumers who consume organic products and propose a research model that can be tested through quantitative studies in the future.

## Figures and Tables

**Table 1:** Demographics.

Variable	Description	fi	%
City	Lima	710	100%
Education level	Graduate	422	59.44%
	Undergraduate	288	40.56%
Age	Younger Millennials (23–28 years)	215	30.28%
	Mid-Millennials (29–34 years)	168	23.66%
	Older Millennials (35–44 years)	327	46.06%
Gender	Female	412	58.03%
	Male	298	41.97%
Social class	Upper	18	2.54%
	Middle–Upper	508	71.55%
	Middle	154	21.69%
	Lower–Middle	30	4.22%

**Table 2:** Convergent validity.

Variable	Item	Load Factor	Cronbach’s Alpha	Composite Reliability (CR)	Average Variance Extracted (AVE)
Environmental Awareness	EA2	0.912	0.895	0.916	0.784
	EA3	0.842			
	EA4	0.901			
Green Self-identity	GSI1	0.833	0.770	0.846	0.582
	GSI2	0.654			
	GSI3	0.844			
	GSI4	0.703			
Subjective Norms	SN1	0.606	0.827	0.873	0.638
	SN2	0.901			
	SN3	0.768			
	SN4	0.884			
Environmental Attitudes	EAT1	0.937	0.929	0.937	0.833
	EAT2	0.901			
	EAT3	0.899			
Green Purchase Intention	GPI1	0.712	0.860	0.884	0.660
	GPI2	0.901			
	GPI3	0.705			
	GPI4	0.907			
Total Alpha	0.901				

**Table 3:** Discriminant validity.

	F1	F2	F3	F4	F5	SR AVE
F1	0.784 ^a^					0.885
F2	0.187 **	0.582 ^a^				0.763
F3	0.153 **	0.062	0.638 ^a^			0.799
F4	0.126 **	0.096 *	0.227 **	0.833 ^a^		0.913
F5	0.175 **	0.125 **	0.323 **	0.274 **	0.660 ^a^	0.812

Notes: F1: Environmental Awareness, F2: Green Self-Identity, F3: Subjective Norms, F4: Environmental Attitudes, F5: Green Purchase Intention. F1–F4, F2–F4, F3–F4, F4–F5, and F1–F2, presented bilateral correlation at 0.01 ** level (bilateral) and F2–F3 did not present correlation. ** Significant correlation at the 0.01 level bilaterally, * significant correlation at the 0.05 level bilaterally. ^a^ AVE.

**Table 4:** Results of hypotheses testing.

Hypotheses	Relation	*β*	*p*-Value	Hypotheses
H1	EA-EAT	0.123	0.058	Rejected
H1a	EA-GSI	0.113	**	Accepted
H2	GSI-EAT	0.270	0.016 *	Accepted
H3	SN-EAT	0.199	**	Accepted
H3a	SN-GSI	0.004	0.744	Rejected
H4	EAT-GPI	0.250	**	Accepted

Note: Goodness-of-fit indices: *X*^2^ (gl) = 344.243(129), *X*^2^/g = 2.669, NFI = 0.960, TLI = 0.969, CFI = 0.974, RMSEA = 0.048. ** The correlation is significant at the 0.01 level (bilateral). * The correlation is significant at the 0.05 level (bilateral). Source(s): Authors’ work.

## Data Availability

The data presented in this study are available in: https://drive.google.com/drive/folders/14gvWexlFmeRpDIk5BW8MYg8B06AAzj72?usp=sharing (accessed on 16 November 2023).

## Appendix A. Survey Questions

**Item**	**Question**	**Source**
EA1	I am very concerned about the environment.	[68]
EA2	Major political change is needed to protect the natural environment.	
EA3	Major social changes are needed to protect the natural environment.	
EA4	Anti-pollution laws should be enforced more vigorously.	
GSI1	I think of myself as someone who is concerned about environmental issues	[48]
GSI2	I think of myself as a ‘green’ consumer	
GSI3	Buying this chair would make me feel like a green consumer	
GSI4	I would feel totally satisfied with myself if I bought this chair	
SN1	Most of my friends think purchasing	[4]
SN2	Most of my colleagues think purchasing organic products is the right thing to do	
SN3	Most of my family members think purchasing organic products is the right thing to do	
SN4	My acquaintances would approve my decision to buy organic products	
EAT1	I think organic products help save nature and its resources	
EAT2	Environmental protection is important to me when shopping for products	
EAT3	I have a favorable attitude toward purchasing organic products	
EAT4	If I get a choice, I’ll prefer an organic product over a conventional product	
GPI1	I consider purchasing organic products because they are less polluting	
GPI2	I consider switching to other brands for ecological reasons	
GPI3	I intend to buy organic products	
GPI4	I intend to switch to an organic version of a product	
